# Systemic immunosuppression from ultraviolet radiation exposure inhibits cancer immunotherapy

**DOI:** 10.1136/jitc-2025-012527

**Published:** 2025-10-31

**Authors:** Pedro Durao, Timothy Budden, Karthik Mallela, Martha Gutteridge, Isabella Mataloni, Shilpa Gurung, Mirco Mastrangelo, Luke Roberts, Antonia Banyard, Garry Ashton, Graham M Lord, Adam Hurlstone, Sara Valpione, Amaya Viros

**Affiliations:** 1 Skin Cancer and Ageing Lab, Cancer Research UK Manchester Institute; The Oglesby Cancer Research Building, The University of Manchester, Manchester, UK; 2 Division of Immunology, Immunity to Infection and Respiratory Medicine, The University of Manchester, Manchester, UK; 3 Cancer Research UK Flow Cytometry Facility, Cancer Research UK Manchester Institute; The Oglesby Cancer Research Building, The University of Manchester, Manchester, UK; 4 Cancer Research UK Histology Facility, Cancer Research UK Manchester Institute; The University of Manchester, Manchester, UK; 5 Centre for Gene Therapy and Regenerative Medicine, School of Basic and Medical Biosciences; Faculty of Life Sciences and Medicine, King’s College London, London, UK; 6 Division of Infection Immunity and Respiratory Medicine, School of Biological Sciences, Faculty of Biology, Medicine and Health, The University of Manchester, Manchester, UK; 7 The Christie NHS Foundation Trust, Manchester, UK; 8 Division of Cancer Sciences, University of Manchester, Manchester, UK; 9 National Biomarker Center, Cancer Research UK, Manchester, UK; 10 NIHR Manchester Biomedical Research Centre, Manchester, UK; 11 Department of Dermatology, Salford Royal NHS Foundation Trust, The University of Manchester, Manchester, UK

**Keywords:** Immune Checkpoint Inhibitor, Colorectal Cancer, Skin Cancer, T regulatory cell - Treg

## Abstract

**Background:**

Ultraviolet radiation (UVR) affects local cutaneous and systemic immunity acutely. The wavelength, pattern and intensity of UVR exposure, individual skin phototype and immune state of individuals modulate the impact of UVR systemically. Local cutaneous immunity after UVR leads to immunosuppression that impacts melanoma. However, the effects of systemic UVR-induced changes on solid cancer therapy are not known.

**Methods:**

We investigated the impact of repeated UVR exposure on systemic immunity and immune checkpoint blockade (ICB) tumor responses in colorectal cancer and melanoma mouse models.

**Results:**

Animals exposed to chronic UVR exhibit decreased ICB response, which is mediated by systemic factors. Repeated UVR exposure expanded systemic lymphocyte populations and contracted total systemic myeloid cells. Specifically, UVR expanded peripheral blood CD4^+^ regulatory T cells (Tregs), which in turn led to greater Treg infiltration and immunosuppression in colorectal and skin cancer, and colorectal ICB-resistant tumors expressed unique pathways of ICB resistance due to systemic UVR. The response to ICB was restored with systemic pharmacological depletion of Tregs. In preliminary human data, there is an association between the molecular evidence of repeated UVR exposure in dermal fibroblasts and higher systemic Tregs.

**Discussion:**

Our data indicate that patients with melanoma and other cancers on immunotherapy should avoid repeated sun exposure.

**WHAT IS ALREADY KNOWN ON THIS TOPIC**

- Ultraviolet radiation (UVR) alters cutaneous and systemic immunity, but its influence on immunotherapy outcomes in solid cancers remains unclear.

**WHAT THIS STUDY ADDS**

- We show that systemic UVR exposure increases circulating regulatory T cells (Tregs) and induces an immunosuppressive signature within colorectal and melanoma mouse tumors, thereby impairing the efficacy of immunotherapy. Humans with cutaneous molecular hallmarks of UVR exposure have higher circulatory Tregs.

**HOW THIS STUDY MIGHT AFFECT RESEARCH, PRACTICE OR POLICY**

- These findings suggest that patients may benefit from avoiding sun exposure during immunotherapy.

## Introduction

 Epidemiological and experimental evidence demonstrate that ultraviolet radiation (UVR) exposure increases the incidence of melanoma and non-melanoma skin cancers.^1^,^4^ The role of UVR in skin cancer initiation has been linked to direct mutational damage^5^ as well as to acute tumor-promoting inflammation in the skin.^6 7^ Additionally, chronic UVR exposure degrades the dermal extracellular matrix^8 9^ and modifies cutaneous skin, leading to local immunosuppression, both of which impact melanoma invasion, immune editing, patient survival and therapy responses.^10^,^13^

In addition to the local, cutaneous effects of UVR exposure, UVR impacts systemic immunity immediately after sun exposure and long-term.^14 15^ Previous work shows that UVR leads to both local, cutaneous and systemic immunosuppression, and systemic factors reduce antitumor immunity and support tumor growth.^16 17^ Furthermore, local, cutaneous immunosuppression after acute UVR reduces ICB response in animals with cutaneous melanoma due to neutrophil expansion.^13^ However, it is likely the role UVR plays extends to other cell types in the skin and systemically, as evidence shows that acute UVB exposure in mice expands CD4^+^ regulatory T cells (Tregs) in the skin.^18^ Therefore, we hypothesized that chronic, repeated UVR exposure (ultraviolet wavelength A, B, UVA/UVB) could impact peripheral immunity, the systemic immune cell landscape, cancer therapy and the pathways of therapy resistance mediated by systemic factors.

We investigated the effects of repeated UVR exposure on systemic immunity, cancer growth and the response to immune checkpoint blockade (ICB) in models of colorectal cancer and melanoma implanted at UVR-protected sites. We found that repeated UVR exposure changes systemic immunity in vivo, and UVR-driven systemic factors inhibited immunotherapy response. UVR exposure expanded peripheral blood Tregs and increased Tregs infiltration in colorectal and skin cancers, leading to reduced ICB response with anti-programmed cell death protein-1 (PD-1). Tregs recruited and activated in tumors mediate cancer immune evasion,^19^ and we found that the pharmacological depletion of Tregs after UVR exposure restored the response to ICB with anti-PD-1.

## Results

### Chronic UVR exposure impacts peripheral immunity

To study whether repeated UVR exposure impacts systemic immunity, cancer growth and immunotherapy response, we first exposed half the back of immunocompetent C57BL/6J mice to 6 standard erythema doses (SED) of UVR each week for 1, 6, 10 or 20 total consecutive weeks, exposing the same half as previously established.^5^ We chose a weekly UVR regime after determining that mild cutaneous inflammation after 6SED resolved on day 7 (one dose of UVR, online supplemental figure 1A–F) and reproduced the hallmarks of the human response to mild sunburn.^5^

**Figure 1:**
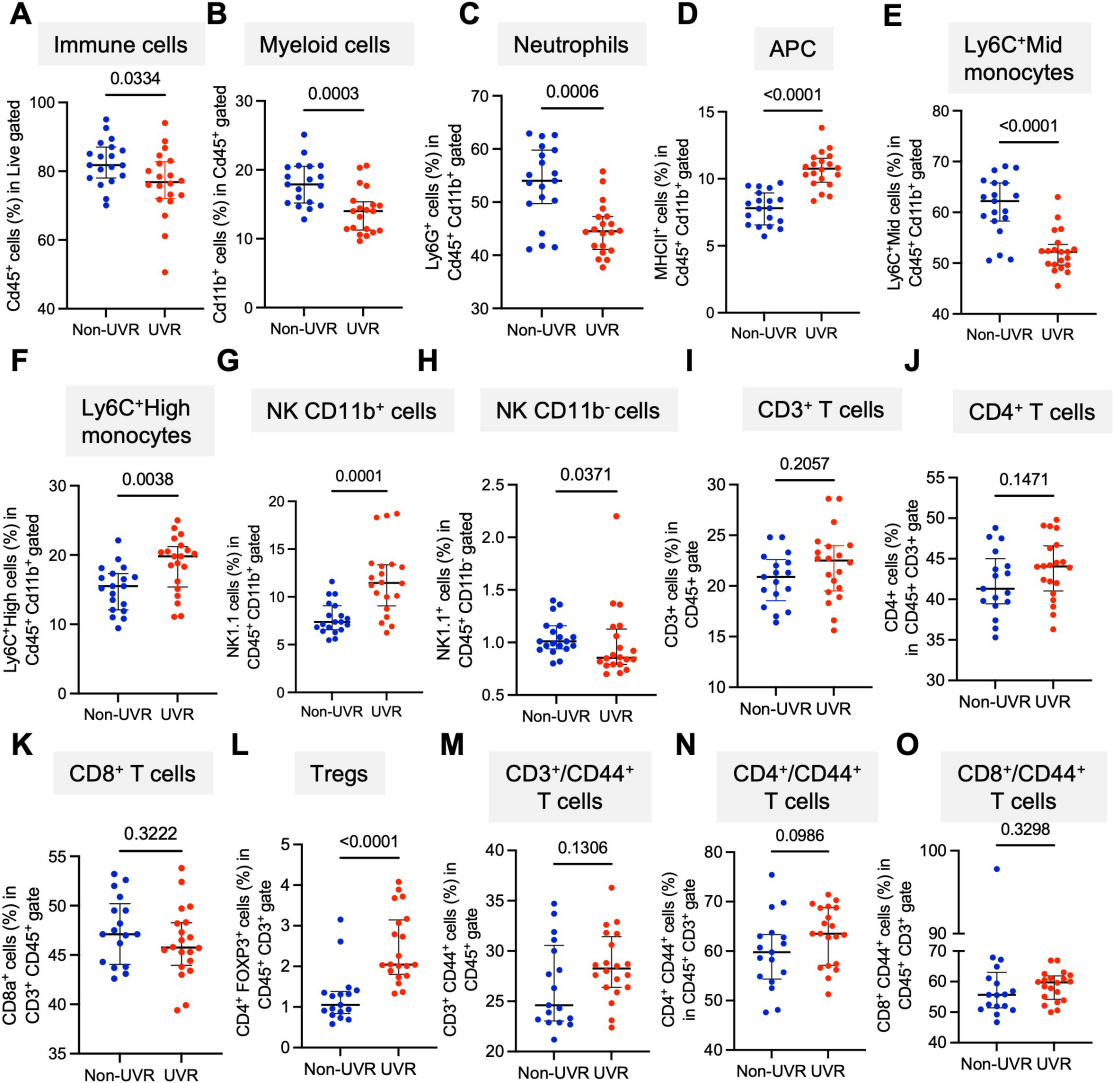
Chronic UVR exposure impacts peripheral immunity. (**A**) total CD45^+^ cells (**B**) CD11b^+^ myeloid cells (**C**) Ly6G+cells in CD11b^+^ gate, (**D**) MHC-II^+^ cells in CD11b^+^ gate, (**E**) Ly6C^+^Mid cells in CD11b^+^ gate, (**F**) Ly6C^+^High cells in CD11b^+^ gate, (**G**) NK1.1^+^ cells in CD11b^+^ gate, (**H**) NK1.1^+^ cells (%) in CD11b^−^ gate. All immune populations determined by flow cytometry in the peripheral blood of UVR mice (n=20) exposed to 6 weeks of UVR and non-UVR (n=19) matched controls. (**I**) CD3^+^ cells, (**J**) CD4^+^ cells in CD3^+^ gate, (**K**) CD8a^+^ cells in CD3^+^ gate, (**L**) CD4^+^FOXP3^+^ cells in CD3^+^ gate, (**M**) CD44^+^ cells in CD3^+^ gate, (**N**) CD4^+^CD44^+^ cells in CD3^+^ gate, (**O**) CD8a^+^CD44^+^ cells CD3^+^ gated immune cell populations by flow cytometry in the peripheral blood of UVR mice exposed to 10 weeks of UVR (n=20) and non-UVR (n=17) matched controls. All values are percentage gated from live and CD45+cells. Whisker graphs depict individual values, medians and IQRs, Mann-Whitney two-tailed tests. APC, antigen-presenting cells; MHC, major histocompatibility complex; NK, natural killer; UVR, ultraviolet radiation.

Acute UVR exposure impacts systemic immunity,^14^ so we tested how repeated exposure to UVR, delivered weekly to animal skin, impacts the peripheral immune cell landscape when sunburn effects subside, 6 weeks and 10 weeks after UVR exposure. First, we compared the immune cell compartment in peripheral blood of UVR and non-UVR animals 7 days after the last of 6 weeks of UVR exposure. This revealed a systemic decrease in overall immune (CD45^+^) and myeloid cells (CD45^+^/CD11b^+^) (figure 1A,B, extended figure 2) in UVR animals. Further characterization of the myeloid compartment showed that the peripheral blood of UVR animals contracted populations of neutrophils (CD45^+^/CD11b^+^/Ly6G^+^), Ly6C^+^Mid monocytes (CD45^+^/CD11b^+^/Ly6C^+^Mid) and NK CD11b^−^ cells (CD45^+^/CD11b^−^/NK1.1^+^). In contrast, antigen-presenting cells (APCs, CD45^+^/CD11b^+^/MHC-II^+^), Ly6C+High monocytes (CD45^+^/CD11b^+^/Ly6C^+^High) and NK CD11b^+^ cells (CD45^+^/CD11b^+^/NK1.1^+^) expanded with long-term UVR exposure (figure 1C–H, online supplemental figure 2). Next, we compared the CD3^+^ cell compartment in peripheral blood of UVR and non-UVR animals 7 days after the last of 10 UVR weekly treatments, which showed a trend for CD3^+^ T cells to expand after chronic UVR (figure 1I–O). Importantly, UVR animals had a significant expansion in CD3^+^/CD4^+^/FOXP3^+^ Tregs (figure 1L) which persisted after 20 weeks of UVR exposure (online supplemental figure 3A–E).

**Figure 2:**
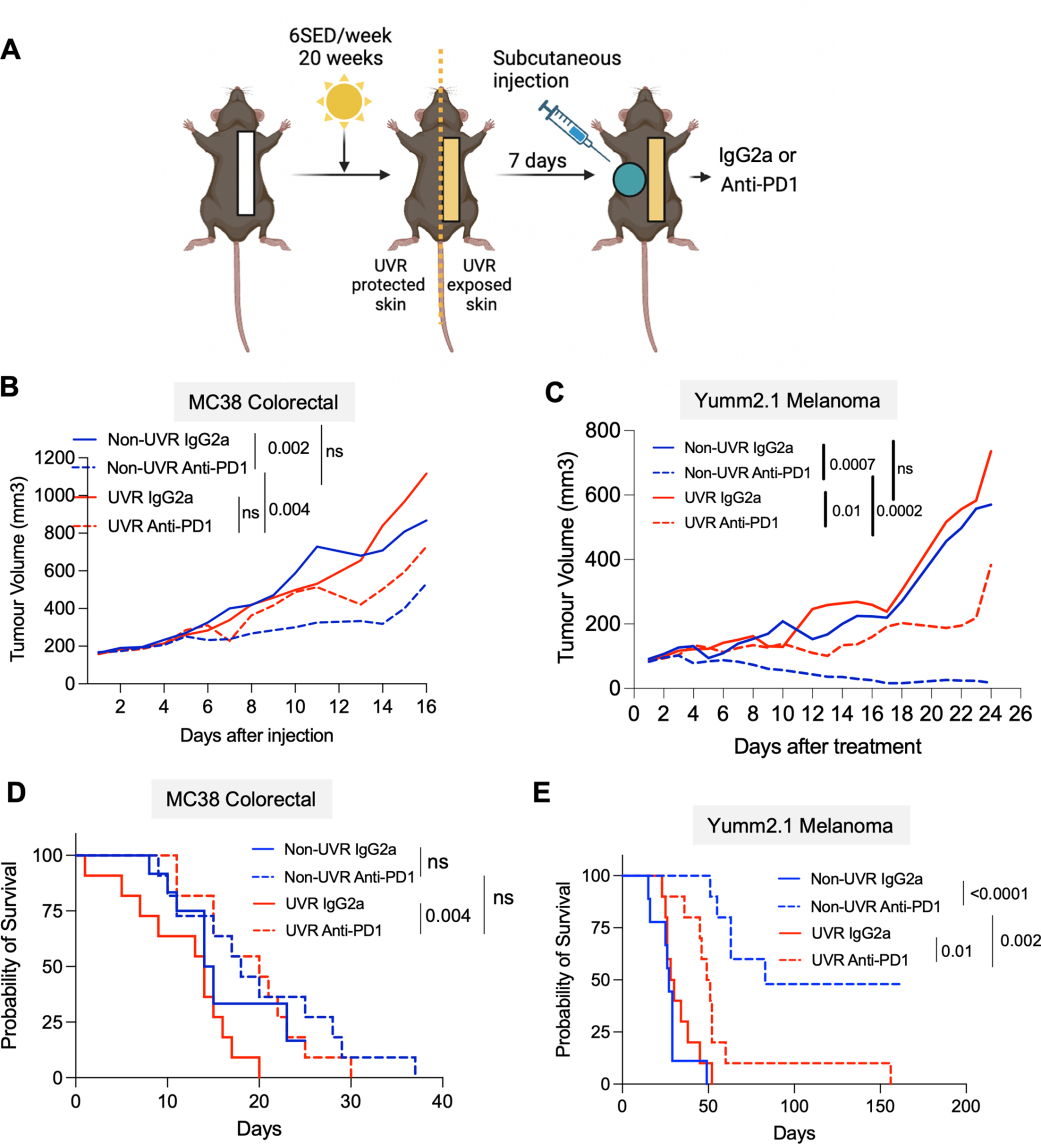
Systemic UVR inhibits immunotherapy response in colorectal cancer and melanoma skin cancer. (**A**) Experimental model. (**B**) Tumor growth of MC38 colorectal and (**C**) Yumm2.1 melanoma cells injected subcutaneously into UVR-protected flanks non-UVR and UVR animals treated with IgG2a (non-UVR IgG2a, UVR IgG2a) and anti-PD-1 (non-UVR anti-PD-1, UVR anti-PD-1), Tukey’s multiple comparison tests. (**D**) Kaplan-Meier survival of non-UVR IgG2a, UVR IgG2a, non-UVR anti-PD-1, UVR anti-PD-1 animals injected with MC38 and (**E**) Yumm2.1 cells; survival Mantel Cox test. MC38: non-UVR IgG2a: n=12; MC38: non-UVR anti-PD-1: n=11; MC38 UVR IgG2a: n=12; MC38 UVR anti-PD-1: n=11; Yumm2.1 non-UVR IgG2a: n=9; Yumm2.1 non-UVR anti-PD-1: n=10; Yumm2.1 UVR IgG2a: n=10; Yumm2.1 UVR anti-PD-1: n=10. PD-1, programmed cell death protein-1; UVR, ultraviolet radiation; 6SED, 6 standard erythema doses.

### Systemic UVR inhibits immunotherapy response in colorectal cancer and melanoma skin cancer

To investigate whether UVR-driven peripheral immune changes affect cancer growth and immunotherapy efficacy, we injected 5,000,000 Yumm2.1 melanoma or 100,000 MC38 colorectal cancer cells into the UVR-protected, contralateral subcutaneous flanks of 20-week UVR-treated and matched non-UVR animals, 7 days after the last of 20 UVR exposures on week 21 (figure 2A). This model allowed us to assess the effects of systemic UVR-driven changes on tumor growth and therapy, as tumor onset and development occurs at UVR-protected sites. This revealed that systemic factors following UVR exposure did not significantly affect the rate of tumor growth (non-UVR IgG2a, UVR IgG2a, figure 2B,C, online supplemental figure 3F,G).

Next, we tested whether systemic UVR-driven changes affect immunotherapy response to anti-PD-1 in colorectal cancer and melanoma (figure 2A). When tumor volumes reached 80–150 mm^3^ (Yumm2.1) and 150–200 mm^3^ (MC38), we initiated ICB treatment with anti-PD-1, which revealed that chronic UVR exposure reduced anti-PD-1 response in both Yumm2.1 and MC38 tumors (non-UVR anti-PD-1, UVR anti-PD-1, figure 2B,C, online supplemental figure 3F–I). Thus, UVR anti-PD-1 animals presented larger tumor volumes compared with non-UVR anti-PD-1 animals at the time of sacrifice. Yumm2.1 anti-PD-1 UVR animals had decreased survival compared with anti-PD-1 non-UVR, a trend that was also visible in the MC38 animals (figure 2D,E, online supplemental figure 3F–I), where animals started therapy with larger tumor volumes.

### Systemic UVR drives immunosuppressive signature in tumors

**Figure 3:**
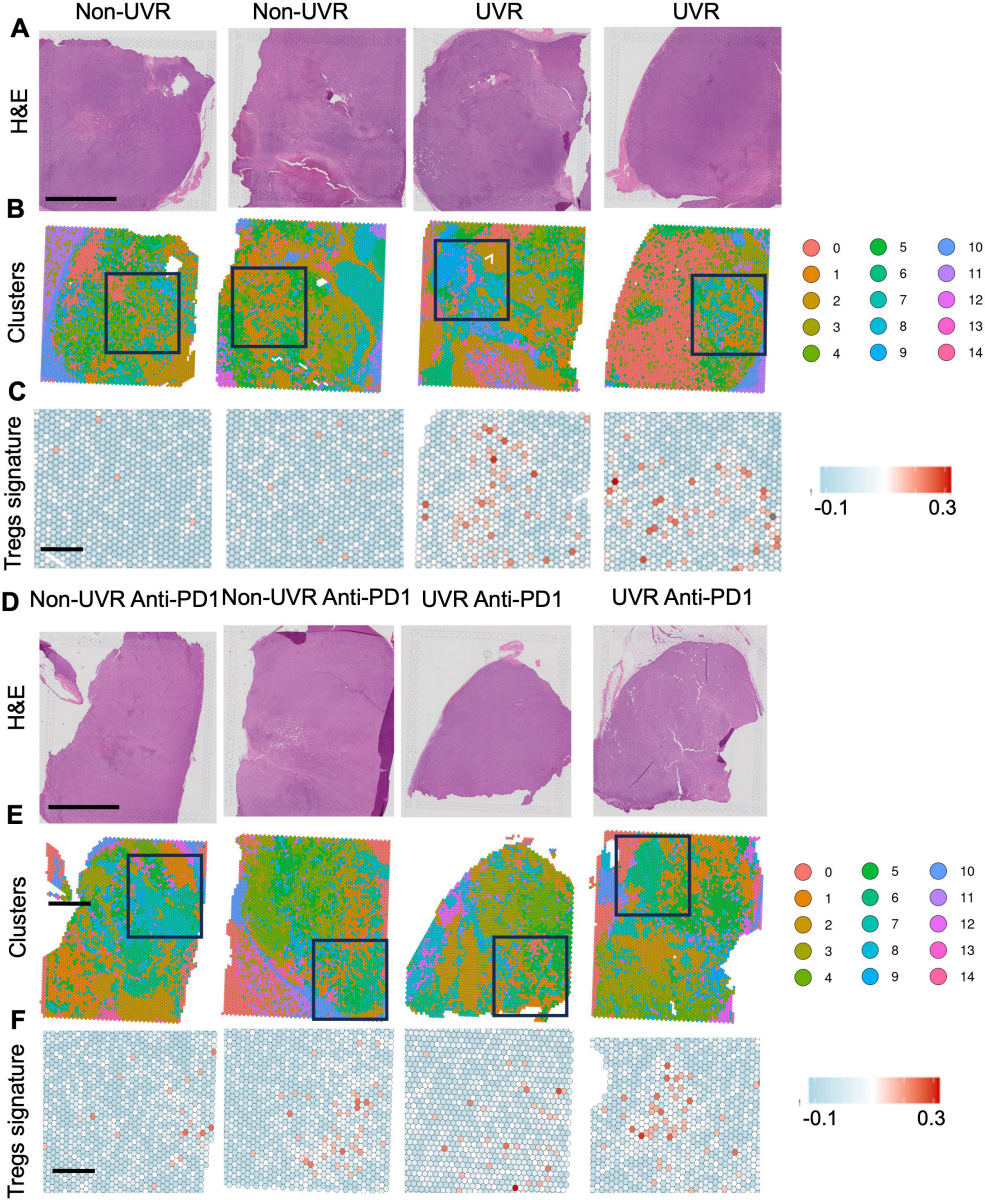
Systemic UVR drives immunosuppressive signature in tumors. (**A**) H&E sections of non-UVR (n=2) and UVR (n=2) MC38 tumors. Scale bar 2.5 mm. (**B**) Spatial transcriptomic of MC38 tumors from A. Color map of gene expression-based clusters representing distinct spatial transcriptional signatures and cell types in all non-UVR and UVR MC38 spatially analyzed tumors. Black box is an area for representative immunosuppressive signature representation. (**C**) Expression of immunosuppressive signature associated with Tregs in non-UVR and UVR MC38 tumors from A, red: high expression, blue: low expression, scale bar=500 µm (**D**) H&E sections of MC38 non-UVR anti-PD-1 (n=2) and MC38 UVR anti-PD-1 (n=2) tumors. Scale bar 2.5 mm. (**E**) Spatial transcriptomic of MC38 anti-PD-1 tumors from D color map of gene expression-based clusters representing distinct spatial transcriptional signatures and cell types in all non-UVR and UVR MC38 spatially analyzed tumors. Black box is an area for representative immunosuppressive signature representation. (**F**) Expression of immunosuppressive signature associated with Tregs in MC38 non-UVR anti-PD-1 and MC38 UVR anti-PD-1 tumors from D, red: high expression, blue: low expression, scale bar=500 µm. PD-1, programmed cell death protein-1; Treg, regulatory T cells; UVR, ultraviolet radiation.

**Figure 4:**
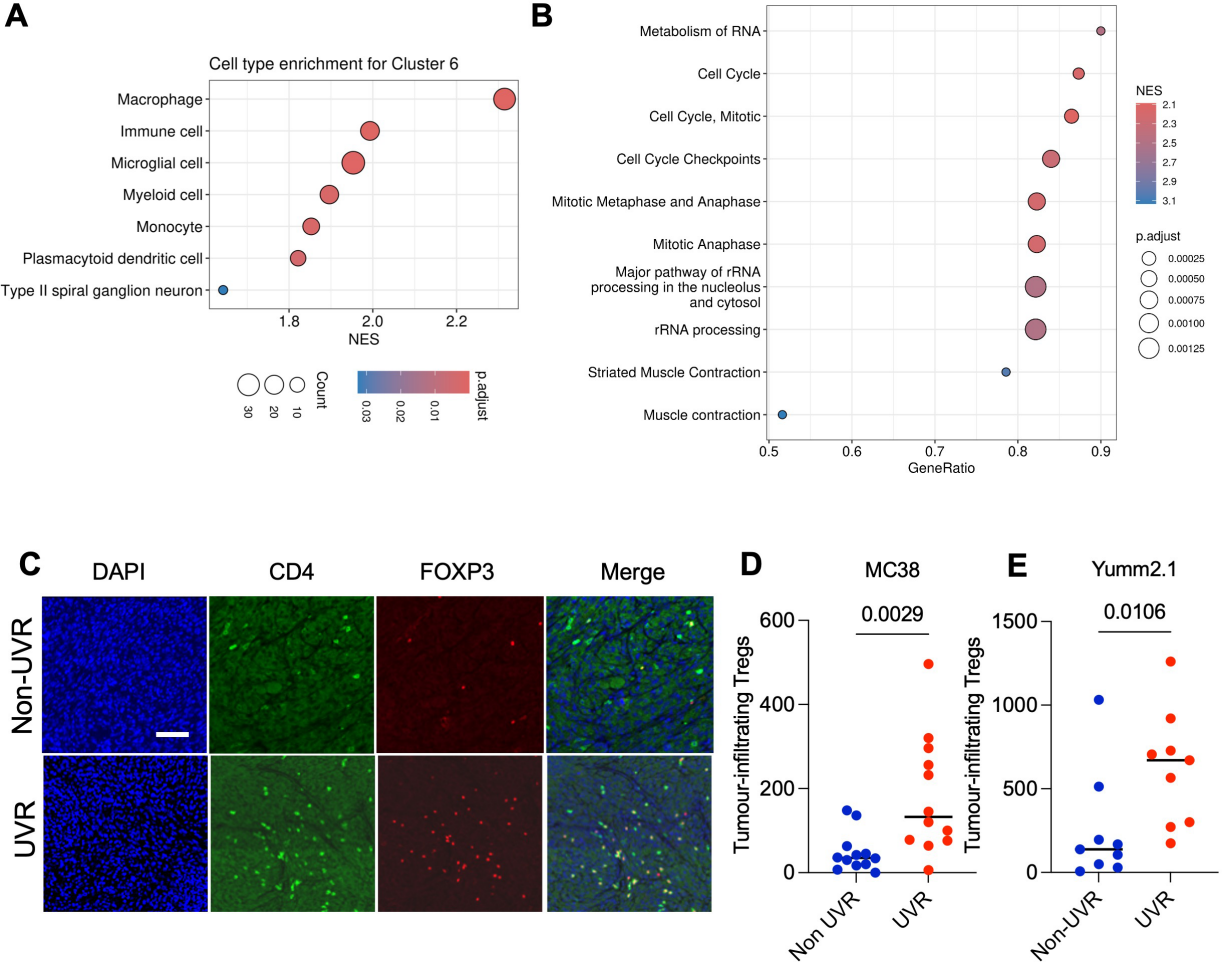
Systemic UVR drives the immunosuppressive tumor immune microenvironment. (**A**) Dot plot cell types enriched cluster 6 based on marker genes from spatial transcriptomic analysis Gene Set Enrichment Analysis. Spot color represents adjusted p value, size represents count of marker genes in cell type classifier, x-axis represents NES. (**B**) Dot plot of top 10 pathways from spatial transcriptomic analysis Gene Set Enrichment Analysis of genes differentially expressed between MC38 non-UVR and UVR tumors. Spot size represents adjusted p value, color represents NES, positive values: enriched in non-UVR tumors. (**C**) Immunofluorescence staining of Tregs in representative non-UVR and UVR mouse tumor bodies. DAPI (blue), CD4^+^ (green) and FOXP3^+^ (red), scale bar 100 µm. (**D**) Immunohistochemistry count of FOXP3^+^ cells in MC38 tumors (**E**) and Yumm2.1 in non-UVR (n=21) and UVR (n=21) mice. Mann-Whitney two-tailed tests. DAPI, 4’,6-diamidino-2-phenylindole; NES, normalized enrichment score; Treg, regulatory T cells; UVR, ultraviolet radiation.

To explore how UVR-driven systemic changes impact tumor immunity with and without anti-PD-1 immunotherapy, we compared size-matched MC38 subcutaneous tumors harvested from the UVR-protected flanks of UVR (UVR IgG2a control n=2, UVR anti-PD-1 n=2) and non-UVR animals (non-UVR IgG2a control n=2, non-UVR anti-PD-1 n=2; figure 3). We performed spatial transcriptomics to capture the tissue heterogeneity at high resolution. Clustering analysis revealed 14 distinct transcriptional clusters present across both non-UVR and UVR MC38 tumors (figure 3, onlinesupplemental figure 3J  table 1). Top gene expression markers show that these clusters represent both unique cell types including fibroblasts (cluster 7: *Col12a1*, *Dpt*, *Pdgfra*), myeloid cells (cluster 6: *H2-Eb1*, *H2-Aa*, *CD74*), and endothelial cells (cluster 9: *Cdh5*, *Plvap*), as well as signatures that may represent separate tumor transcriptional programs such as hypoxia (cluster 2: *Hspa1b*, *Hmox1*, *Ndrg1*) and interferon response (cluster 8: *Irf7*, *Ifi47;* onlinesupplemental figure 3J  table 1). The frequency of these clusters varies by treatment conditions and history of UVR (online supplemental figure 4A–D), and we identified specific cluster enrichments in tumors in UVR mice. For example, cluster 12, identified by high expression of the histone H2A isoform (Hist2h2ac), which is associated with epithelial to mesenchymal transition,^20^ was fourfold higher in the UVR anti-PD-1 tumors compared with non-UVR anti-PD-1 tumors (figure 3, onlinesupplemental figure 4AD and table 2). To identify biological changes in tumors in non-UVR and UVR mice, we next ran pseudo-bulk differential gene expression analysis. Focusing on UVR and non-UVR control tumors (IgG2a), we found MC38 tumors from UVR animals were enriched in messenger RNAs (mRNAs) that are critical for Tregs regulation and immunosuppressive function compared with non-UVR tumors (online supplemental table 3). Additionally, MC38 tumors from UVR control (IgG2a) animals had significantly higher levels of the main Tregs homing molecule *Ccr5*^21 22^ and of *Il15*,^23 24^ a cytokine that has been described as critical in promoting dermal Tregs proliferation, compared with non-UVR controls. Moreover, MC38 tumors from UVR mice had increased expression of genes involved in the tumor growth factor (TGF) (*Tgfbr1*, *Tgfb2*, *Tgfb3*, *Tgfbr3*) and interleukin 10 (*Il10ra* and *Il10rb*) immunosuppressive pathways that are critical for Treg immunosuppressive function,^25^ along with an increased expression of the exhaustion^26^ and adenosine-driven immunosuppression marker *Entpd1*^27^ (online supplemental table 3). To spatially compare the expression of immunosuppressive genes, we generated an immunosuppressive gene signature for these markers to define a Treg-driven immunosuppressive environment and found that this was significantly increased in the UVR tumors (figure 3C, online supplemental figure 4E). Examining the immunosuppressive signature by cluster revealed enrichment of this signature in a cluster enriched for myeloid cell markers (cluster 6, figures3B  4A, onlinesupplemental tables 3  4). This suggests that myeloid cells in UVR-treated mice could drive a more immunosuppressed microenvironment, with more Tregs, in tumors developing at UVR-protected sites in UVR mice compared with tumors in non-UVR mice. Additionally, Gene Set Enrichment Analysis (GSEA) of differentially expressed genes showed UVR tumors highly expressed cell cycle and RNA metabolism pathway genes compared with non-UVR tumors (figure 4B, online supplemental table 5). Taken together, these findings indicate that systemic effects of UVR exposure impact the tumor immune microenvironment (TIME), driving increased Tregs and extracellular adenosine immunosuppressive functions. The colorectal and melanoma tumors grew at UVR-protected sites in UVR-exposed animals, indicating that changes in the TIME are due to systemic immune modulation induced by repeated cycles of UVR exposure.

**Figure 5:**
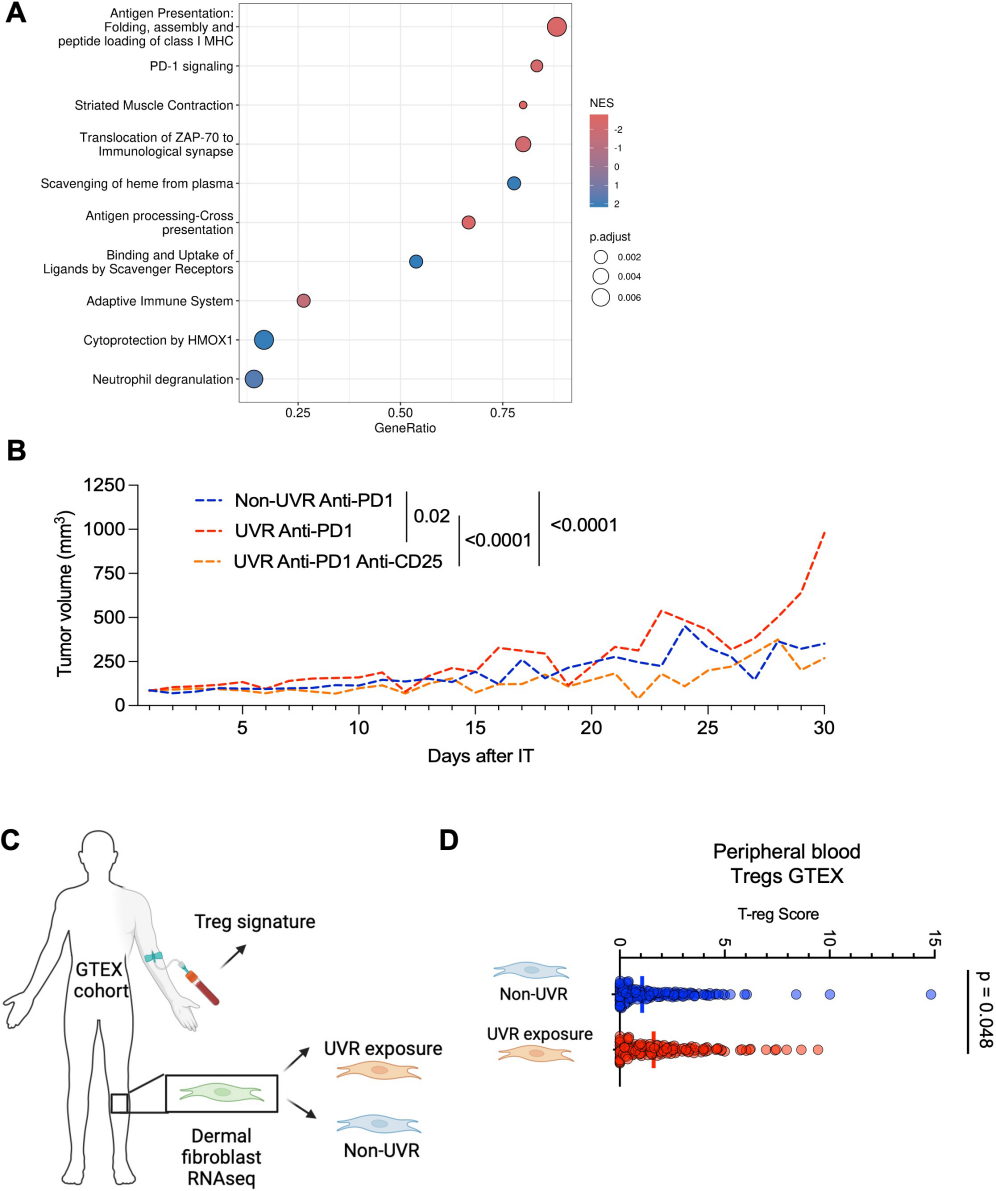
Treg inhibition partially restores anti-PD-1 response in UVR animals. (**A**) Dot plot of top 10 pathways from spatial transcriptomic analysis Gene Set Enrichment Analysis of genes differentially expressed between MC38 non-UVR anti-PD-1 and UVR anti-PD-1 tumors. Spot size represents adjusted p value, color represents NES, positive: enriched in non-UVR tumors, negative: enriched in UVR tumors. (**B**) Tumor growth of Yumm2.1 cells injected subcutaneously into non-UVR anti-PD-1 (n=7, UVR anti-PD-1 (n=5), UVR anti-PD-1 anti-CD25 animals (n=8), Tukey’s multiple comparison tests. (**C**) Cartoon showing experimental approach for classifying UVR signature in GTEx patient cohort. (**D**) Tregs score from the peripheral blood of non-UVR (n=143) and UVR (n=142) GTEx patients, Mann-Whitney one-tailed test. GTEx, Genotype-Tissue Expression; MHC, major histocompatibility complex; NES, normalized enrichment score; PD-1, programmed cell death protein-1; RNA-seq, RNA sequencing; Treg, regulatory T cells; UVR, ultraviolet radiation.

We confirmed that Tregs are more abundant in the expanded cohorts of subcutaneous MC38 and Yumm2.1 tumors of UVR mice compared with non-UVR control animals with immunofluorescence (CD4^+^ FOXP3^+^) and immunohistochemistry (FOXP3^+^) (figure 4C–E). The higher infiltration in UVR tumors supports that repeated UVR exposure expands systemic Tregs in peripheral blood, and Tregs infiltrate tumors at sun-protected sites to establish an immunosuppressive TIME. We did not observe significant changes in other immune cell populations, such as neutrophils, macrophages and natural killer (NK) cells (onlinesupplemental figure 5AC and table 6).

Animals exposed to UVR present control tumors with increased Tregs, and the spatial transcriptomics analysis suggests an immunosuppressive TIME driven by Treg function, so we hypothesized that increased Tregs and Treg-dependent TIME immunosuppression are a possible mechanism of therapy evasion in tumors that do not respond to anti-PD-1 in UVR animals. Examining the immunosuppressive gene signature across samples revealed that UVR anti-PD-1 MC38 resistant tumors were indeed enriched for the critical Treg regulation and immunosuppressive function signature (UVR anti-PD-1, figure 3D–F, onlinesupplemental figure 4E  table 2), like treatment-naïve, MC38 tumors in UVR control animals (figure 3E,F, onlinesupplemental figure 4E  table 3). MC38 UVR anti-PD-1 resistant tumors, like UVR controls, had significantly higher levels of *Ccr5*,^21 22^ *Il15*,^23 24^ tumor growth factors (*Tgfbr1*, *Tgfb2*, *Tgfb3*, *Tgfbr3*), *Il10ra* and *Il10rb*, and higher *Entpd1*^27^ compared with MC38 non-UVR anti-PD-1 resistant tumors (figure 3F, onlinesupplemental figure 4E  table 3). These data suggest that tumors with high Tregs leading to increased immunosuppressive TIME retain this signature during immunotherapy primary resistance. Furthermore, GSEA of genes differentially expressed between anti-PD-1 treated non-UVR and UV tumors revealed that UVR anti-PD-1 resistant tumors expressed additional immune pathways. Specifically, UVR anti-PD-1 resistant tumors were enriched for antigen presentation, PD-1 signaling, antigen processing and cross-presentation, and adaptive immune signaling pathways, while non-UVR tumors were enriched for oxidative stress response and neutrophil degranulation (figure 5A, online supplemental table 7). This aligns with existing literature identifying PD-1 as a hallmark of T-cell exhaustion, contributing to immunosuppression and the maintenance of self-tolerance.^28^ In the context of our spatial transcriptomics findings, which reveal the presence of a Treg-associated immunosuppressive signature and Treg infiltration in UVR anti-PD-1-resistant tumors, elevated PD-1 signaling indicates an ineffective T-cell response leading to impaired tumor clearance. Furthermore, the upregulation of neutrophil degranulation and oxidative stress in non-UVR tumors is coherent with the protumoral and pro-migratory function of neutrophil extracellular traps.^29^ Taken together, these findings indicate that systemic effects of UVR exposure impact the TIME, driving increased tumor Tregs and unique immune pathways linked to immunotherapy resistance.

### Treg inhibition partially restores anti-PD-1 response in UVR animals

We next explored whether elevated Tregs in chronically UVR-exposed animals are responsible for impaired immunotherapy response and hypothesized that Treg depletion after UVR would restore immunotherapy response. For this, we initiated Yumm2.1 tumors in UVR animals and treated them with anti-PD-1 and anti-CD25, an antibody that depletes systemic CD25^+^ cells which are primarily present at high levels in Tregs, which can modestly reduce tumor growth by depleting Tregs in murine melanoma models.^30^ Importantly, we found that Treg depletion with anti-CD25 reduced systemic Foxp3+ cells, and critically, restored the response to anti-PD-1 in animals with UVR exposure (figure 5B, online supplemental figure 5D). Finally, Treg depletion with anti-CD25 led to increased numbers of Cd8+cells at the leading edge of the tumors (online supplemental figure 5E,F).

To confirm our findings are relevant to UVR-exposed humans, we examined whether individuals from the Genotype-Tissue Expression (GTEx) cohort, who present molecular evidence of chronic UVR exposure in the skin,^8 10^ have more Tregs in peripheral blood. To classify UVR exposure in the skin, we used a surrogate composite RNA expression score present in the dermal lower limb fibroblasts, which is imposed by UVR extracellular matrix dermal degradation programs previously shown to be upregulated in human skin that is chronically sun exposed^10^ (figure 5C). Within the GTEx database, cutaneous calf fibroblasts and matched peripheral whole blood gene expression data were available for 428 individuals. We analyzed the expression hallmarks of UVR-induced dermal fibroblasts using a chronic sun damage score (CSD score),^10^ and patients were stratified into CSD terciles. We compared the immune cell profile in peripheral blood of patients in the lower (n=143 individuals, median CSD score=179.4092) and top (n=142, median=332.2376) terciles, removing the intermediate group to generate clear categories of UVR exposure. This comparison revealed that the proportion of Treg cell signature in the peripheral blood of individuals stratified in the group with higher CSD had an increased proportion of Tregs in peripheral blood compared with individuals in the lower CSD tercile (p=0.048 one-tailed Mann-Whitney; figure 5D). Importantly, no other immune cell types in blood differed between the top and lower terciles. These findings support previous evidence that lymphocyte subsets have a seasonal variation^31 32^ and reveal systemic Tregs expand in individuals who present molecular signature of UVR exposure in the skin fibroblasts (online supplemental figure 6).

## Discussion

Here we show that repeated exposure to UVR over weeks impacts systemic immunity, leading to changes in T-cell subsets and contraction of total myeloid cells in peripheral blood. Specifically, there is a significant increase in Tregs, a lower proportion of Ly6C^+^Middle monocytes, neutrophils and NK/CD11b^−^ cells, with a proportional increase in Ly6C^+^High monocytes, NK/CD11b^+^ and APCs. We used murine models of colorectal cancer and melanoma to show that systemic immunity affects immunotherapy response. We studied the effect of systemic UVR on immunotherapy by assessing the rate of growth of subcutaneous tumors implanted at UVR-protected sites and the days of animal survival in UVR and non-UVR animals. The in vivo data revealed a loss of ICB therapeutic effect in UVR animals. In the TIME, spatial transcriptomics detected significant differences between UVR (UVR IgG2a, anti-PD-1 UVR) and non-UVR (non-UVR IgG2a, anti-PD-1 non-UVR) animal MC38 tumors. Because the tumors developed independently from UVR (MC38 colorectal cancer murine model and non-UVR genetically engineered melanoma mouse model,^33 34^ and grew at sites protected from UVR-exposed skin, we inferred the changes in the TIME are due to systemic immunity. Tumors growing in UVR animals presented transcriptional signatures including myeloid, antigen-presenting genes and increased stromal cell signatures, including fibroblast, endothelial and immune cell activity genes, which have been linked to poor rates of immunotherapy response.^35^,^37^ Importantly, UVR tumors were more infiltrated by Tregs and were also enriched for mRNAs critical for Treg immunosuppressive function, as well as the Treg homing *Ccr5* and *Il15* molecules, which are key to dermal Treg proliferation and infiltration. Additionally, *TGF*s and *Il10* receptors, as well as the exhaustion and adenosine-driven immunosuppression marker gene *Entpd1,* were increased. Tumor cells and the TIME developed independently of direct UVR exposure, at UVR protected sites, indicating that the systemic effects of UVR exposure modify the TIME, driving increased systemic and local Tregs and immunosuppression. Repeated UVR exposure increases Tregs in the skin^18^ and in peripheral blood,^38 39^ and we show that Tregs expanding in the blood are associated with higher numbers of Tregs in tumors, activation of immunosuppressive pathways and reduced ICB response. Importantly, the pharmacological ablation of Tregs partially restores the response to ICB.

Previous studies have established UVR leads to cutaneous and systemic immunosuppression, and local immunosuppression to the skin impacts melanoma immunotherapy response.^13^ Our work establishes a greater role for UVR in cancer, as we demonstrate systemic effects of long-term UVR exposure leads to systemic immunosuppression, which impacts melanoma and non-melanoma cancer immunotherapy.

Primary ICB resistance in UVR animals manifested with upregulation of other known mechanisms of immunotherapy resistance, such as PD-1 signaling, in addition to increased Tregs and Treg functional gene upregulation. In contrast, ICB-resistant tumors in non-UVR mice upregulated oxidative stress response genes and neutrophil degranulation pathways. These data indicate that systemic UVR impacts therapy resistance. Moreover, the shift we see in the myeloid cell blood compartment following chronic UVR, although not statistically significant in our immunohistochemistry stains, suggests that there may be other UVR-driven immune changes impacting immunity, cancer growth and therapy response, in addition to higher Tregs.

UVR increases the incidence of skin cancer following the injection of syngeneic melanoma cells into UVR-irradiated skin, but not unirradiated skin.^40^ Furthermore, unirradiated mice develop fibrosarcomas when they are joined parabiotically with UVR mice, but not unirradiated animals.^17^ Here, we show that repeated UVR exposure impacts systemic immunity, which leads to an immunosuppressive milieu in the TIME. The UVR-driven systemic changes impact the response to therapy.

Limitations of our study are first, that the depletion of Tregs with anti-CD25 antibody was not orthogonally validated with flow cytometry. We also found the impact on systemic immunity extends beyond Tregs, and ratios of innate immune cells are altered with different doses of accumulated UVR. Additionally, our study suggests that the number of exposures, the dose, pattern and wavelength of UVR will be further variables in the global effect on systemic immunity. It is also possible that UVR impacts the metabolic and functional states of all immune peripheral cells, effecting changes in cancer biology that are independent of the changes we describe in immune cell ratios by UVR. Finally, our human approach to study the link between chronic UVR and systemic Treg elevation uses the molecular signature of UVR damage in calf fibroblasts as a surrogate marker for real life sun exposure. This approach does not adjust for skin types, behavioral habits, technical variability and the heterogeneity in the rate of mutation accumulation, and therefore, the association in humans between UVR exposure and systemic Treg increase remains preliminary and will require further study.

Multiple studies focus on tissue-specific immune factors linked to immunosuppression and ICB resistance.^41 42^ However, this study demonstrates that systemic immunity is a key factor in immunotherapy response in models of skin and colorectal cancer. Critically, we show that systemic immunity is shaped by environmental UVR exposure. Thus, the effect of cutaneous UVR extends from skin cancer initiation to impact cancer immunity via systemic immune changes. Our work suggests that not only patients with melanoma, but other patients with solid cancer receiving immunotherapy may benefit from avoiding sun exposure.

## Materials and methods

### Animal experiments

All mice were maintained in pathogen-free, ventilated cages in the Biological Resources Unit at the Institute, and allowed free access to irradiated food and autoclaved water in a 12 hours light/dark cycle, with room temperature at 21±2°C. All cages contained wood shavings, bedding, and a cardboard tube for environmental enrichment. Female C57BL/6J (4–6 weeks old) were purchased from Charles River and allowed to acclimate for 1-week prior to any procedure.

For UVR treatments, the shaved backs (right flank) of C57BL/6J were shaved the day prior to UVR exposure. All mice were anesthetized with medetomidine (Domitor—1 mg/mL) and Ketamine (Narkaten/Ketamidor—100 mg/mL). Ketamine and medetomidine were mixed and diluted with phosphate buffer saline (PBS) solution to a final concentration of 1 mg/g and 0.01 mg/g of body weight, respectively, and injected subcutaneously. The UVR-exposed cohorts were randomly distributed on a tray and placed under a UVR light source (Waldmann UV6). Animals were covered with a UVR-proof cloth with windows over the shaved areas of the back. Each animal was treated with 60 mJ/cm^2^ (equivalent to 6SED) UVA/UVB once per week each week. Immediately after UVR, mice were injected subcutaneously with atipamezole (5 mg/mL). Cages were placed onto heating pads to prevent extreme body temperature changes during recovery. E45 moisturizer was applied after every UVR session. Sham non-irradiated controls (non-UVR) were exposed to the same anesthetic conditions as the UVR group. Tumor cell injections were performed 7 days after the final UVR session to the contralateral, UVR-protected, non-exposed skin. All cell lines were tested for *Mycoplasma* and mouse hepatitis virus prior to injection into animal flanks.

To study immunotherapy response, two cohorts of animals were injected with cell lines: a control group of mock-irradiated animals (non-UVR; n=42) and an experimental group of UVR animals (n=43). UVR C57BL/6J animals received weekly UVR (6SED) for 20 consecutive weeks on the left flank. 100,000 MC38 or 5,000,000 Yumm2.1 cells were injected subcutaneously into the right, UVR protected, contralateral flanks. Cells were injected 7 days after the final UVR dose, when acute inflammation resolved. Tumor volumes were measured five to six times a week, using the formula (length×width^2^)/2. When Yumm2.1 tumors reached 80–150 mm^3^ and MC38 tumors reached 150–200 mm^3^, anti-PD-1 (2BScientific BE0146; clone RMP1-14; 10 mg/kg) or rat IgG2a (2BScientific BE0089; clone 2A3; 10 mg/kg) were diluted in 100 µL of PBS and administered via intraperitoneal injection every 2–3 days for a total of five doses per mouse: non-UVR+IgG2a+MC38 cells (n=12); non-UVR+anti-PD-1+MC38 cells (n=11); UVR+IgG2a+MC38 Cells (n=12); UVR+anti-PD-1+MC38 cells (n=11); non-UVR+IgG2a+Yumm2.1 cells (n=9); non-UVR+anti-PD-1+Yumm2.1 cells (n=10); UVR+IgG2a+Yumm2.1 cells (n=10); UVR+anti-PD-1+Yumm2.1 cells (n=10). Animals were injected with 5,000,000 Yumm2.1 cells 7 days after the last UVR exposure into the contralateral UVR-protected flanks after 13 weeks of half-back UVR. Tumors were measured three times a week. When tumors reached 80–150 mm^3^, anti-PD-1 (10 mg/kg) was administered via intraperitoneal injection every 2–3 days for a total of five doses per mouse. Anti-CD25 (clone PC-61.5.3; Bio X Cell #BP0012; 20 µg) was administered 1-day prior to and on the day of tumor cell injections, every 5 days for a total of four doses. After the four doses, the dose of anti-CD25 was 200 µg, administered every 3 days until the end of the study. The final cohorts are as follows: non-UVR+anti-PD-1+Yumm2.1 cells (n=7); UVR+anti-PD-1+Yumm2.1 cells (n=5); UVR+anti-PD-1+anti-CD25+Yumm2.1 cells (n=8). Animals were sacrificed when the study endpoint was reached, according to the guidelines set by the Committee of the National Cancer Research Institute as stipulated by AWERB. Full body autopsy including tumor, skin (UVR exposed and non-UVR exposed), brain, heart, lungs, liver, spleen, kidney, and suprarenal tissue was conducted on all mice. Organs were fixed with 10% formalin. Fixed tissue samples were embedded in paraffin and stained with H&E.

To study the acute effect of UVR in the skin (sunburn), the right flank of 5 C57BL/6J mice was irradiated with a single UVR dose. Mice were sacrificed 24 hours, 48 hours, 72 hours, 5 days and 7 days after UVR exposure. Skin from both UVR-protected and UVR-exposed flanks was collected and fixed with 10% formalin. Fixed tissue samples were embedded in paraffin and stained with H&E.

### Peripheral blood collection

Mice were placed in a 37°C heating box for 10 min to allow the tail vein to dilate and 100 µL peripheral blood was collected into EDTA-coated tubes. Red blood cell lysis was performed using ACK Lysing Buffer (Gibco #A1049201) following the manufacturer’s protocol. Blood was collected 7 days after 10 weeks of UVR exposure (lymphoid panel) and 6 weeks of UVR exposure (myeloid panel) for flow cytometry.

### Cell lines

MC38 colon carcinoma (Kerafast ENH204-FP) and Yumm2.1 (Merck #SCC232) melanoma cell lines were cultured in Roswell Park Memorial Institute (RPMI) 1640 medium with L-glutamine (Gibco #11875093), 10% fetal bovine serum (Sigma #F7524) and 1% penicillin-streptomycin (10,000 U/mL) (Gibco #15140122). Cell lines were cultured at 37°C in 5% CO_2_ with medium replaced as required. Cell lines were tested every 6 weeks approximately for *Mycoplasma* using LookOut Mycoplasma PCR Detection Kit (Sigma Aldrich, MP0035) and short tandem repeat (STR) analysis was performed using the American Type Culture Collection (ATCC) Cell Line Authentication Service.

### Flow cytometry

Flow cytometry analysis was performed on the BD LSRFortessa at the Cancer Research UK Flow Cytometry Facility. The following antibodies were used for the analysis of immune populations in the peripheral blood: FITC anti-mouse CD45 (Clone 30-F11; BioLegend 103108; 1:1000 dilution), PE/Cy5 anti-mouse CD3ε (Clone 145–2 C11; BioLegend 100310; 1:200 dilution); BV 421 anti-mouse CD8a (Clone 53–6.7; BioLegend 100738; 1:100 dilution); APC/Fire 750 anti-mouse CD4 (Clone RM4-4; BioLegend 116020; 1:1000 dilution); BV 605 CD44 (Clone IM7; BioLegend 103047; 1:400 dilution); CD11b-PerCP-Cy5.5 (Clone M1/70; BioLegend 101228; 1:500 dilution); Ly6G-PE-Cy7 (Clone 1A8; BioLegend 127618; 1:400 dilution); Ly6C-PE (Clone HK1.4; BioLegend 128008; 1:400 dilution); BV 785 MHC-II (Clone M5/114.15.2; BioLegend 107645; 1:200 dilution); NK1.1-PE-Dazzle (Clone PK136; BioLegend 108748; 1:400 dilution). LIVE/DEAD Fixable Blue Dead Cell Stain (Invitrogen L23105; 1/200 dilution) was used to stain dead cells. All antibodies were diluted in fluorescence-activated cell sorting (FACS) buffer (PBS+2% fetal bovine serum) and samples were incubated with the antibody mix for 45 min at room temperature. After the membrane staining, samples were fixed and permeabilized using eBioscience Foxp3/Transcription Factor Staining Buffer Set (Cat No. # 00-5523-00) following the manufacturer’s protocol. After the fixation and permeabilization process, samples were stained with FOXP3-PE (Clone MF-14; BioLegend 126404; 1:400 dilution). All samples were resuspended in a final volume of 300 µL of FACS buffer. All antibodies were titrated to optimal concentrations prior to use. Compensation beads (Cat No. # 01-3333-42) were used to set up the photomultiplier tubes (PMT) voltages for each antibody and to minimize fluorescence spillover. The gating strategy was designed based on fluorescence minus one controls.

### Spatial transcriptomics

5 µm tumor sections were cut and H&E stained from one mouse in each of the four experimental groups (non-UVR+IgG2a+MC38 cells; non-UVR+anti-PD-1+MC38 cells; UVR+IgG2a+MC38 Cells; UVR+anti-PD-1+MC38 cells). The regions of interest were marked and then selected at microtomy and placed within the active area on the Visium slide. Indexed sequencing libraries were prepared from H&E-stained Visium Spatial Gene Expression Slides using the Visium Spatial for Formalin-Fixed Paraffin Embedded (FFPE) Gene Expression Kit, Mouse Transcriptome, (10x Genomics, 1000339) according to the manufacturer’s protocol. Library quality was checked using the Fragment Analyzer (Agilent). Libraries were quantified by quantitative PCR using a KAPA Library Quantification Kit for Illumina (Roche, 07960336001). Paired-end sequencing with read lengths of 28+10+10+50 bp was performed on a NovaSeq 6000 sequencer (Illumina). The data output was further processed and analyzed in the Seurat package (V.5.0.1) in R (V.4.3.0). The data output was further processed and analyzed in the Seurat package (V.5.0.1) in R (V.4.3.0). All samples were analyzed with the standard Seurat spatial transcriptomics workflow. First, spots with fewer than 100 transcripts detected were removed from all samples and then each sample was normalized with the *SCTransform* function. Samples were merged, and principal component analysis (PCA) was performed on the merged object with the *RunPCA* function. The Harmony package (V.1.0.3) was used to correct for any batch effects between samples. Dimensionality reduction and clustering were performed on the first 30 components, with a clustering resolution of 0.6. Differentially expressed genes between non-UVR and UVR tumors were determined using pseudo-bulk analysis with the *PrepSCTFindMarkers* and *FindMarkers* function with samples grouped by their UVR status as group identifiers after subsetting into IgG and anti-PD-1 tumor groups. Immunosuppressive gene signature was quantified across all tumors using the *AddModuleScore* function for nine immunosuppressive genes identified in differential expression analysis (*Ccr5*, *Tgfb3*, *Il10ra*, *Entpd1*, *Tgfbr1*, *Il10rb*, *Tgfbr3*, *Tgfb2*, *Il15*). GSEA for enriched pathways in differentially expressed genes was performed using the clusterProfiler package (V.4.8.3) and compared with Reactome pathways by converting gene names to Entrez IDs, ranking genes by log fold change and compared with Reactome pathway database using the msigdbr (V.7.5.1), org.Mm.eg.sb (V.3.17.0), and enrichplot (V.1.20.3) packages. Differentially expressed genes from the *FindMarkers* function were filtered by adjusted p value<0.05 and used for GSEA with analysis settings including p value cut-off of 0.1, Benjamini and Hochberg false discovery rate adjustment, minimum gene set size of 5 and maximum 500, with 10,000 permutations. GSEA for cell types based on cluster marker gene expression was compared with the CellMarker database.^43^

### RNA-sequencing data (GTEx)

RNA-sequencing expression file (Gene Transcripts per million, V.8) from the GTEx project was downloaded from the GTEx Portal (https://gtexportal.org/home/), along with de-identified sample and subject data. As there is no clinical data of sun exposure in the GTEx cohort, which includes RNA sequencing of~50+ healthy tissue types from~1,000 deceased human donors, we used a surrogate marker based on genes known to be upregulated by chronic UV exposure in dermal fibroblasts. Some GTEx cases have gene expression data for fibroblasts cultured from their calf skin, so we used these to estimate the patient sun exposure based on the expression of the UV signature in the fibroblasts.

527 patients in the GTEx database have fibroblast samples from the leg. The sun exposure score is calculated as the average expression of the eight genes (MMP1, BMP7, MMP8, MMP3, DCN, APP, BMP2, CTSL) in each sample, which we have previously validated.^10^ This was calculated from the GTEx transcripts per million dataset. 504 fibroblast samples had gene expression data. 755 whole blood samples collected with gene expression and the Immune cell profiles of all GTEx samples were estimated using the CIBERSORTx platform (https://cibersortx.stanford.edu/).^44^ Immune cell fractions were imputed using the LM22 cell signature matrix and 1,000 permutations. 428 samples had both fibroblasts and whole blood gene expression and so could be used for CSD score analysis of the blood immune profile. This group was divided into tertiles based on CSD score, and the lowest and highest groups were compared. This was done to get two distinct groups and remove any intermediate phenotype where CSD may not be clear (middle group). Low CSD had 143 patients and the range of CSD score was 115.461–206.2507 (median=179.4092) and the high CSD score (n=142) had a range of 260.0707–1606.532 (median=332.2376). The analysis was not matched. Once patients were grouped into tertiles based on the UV score of their fibroblast samples, then Treg CIBERSORTx scores were compared in whole blood GTEx samples between the lowest and highest UVR score groups (online supplemental table 8).

### Immunohistochemistry

Rat anti-mouse FOXP3 antibody (Invitrogen #14-5773-82) and 4’,6-diamidino-2-phenylindole (DAPI) (Thermo Fisher 62248) staining was performed according to manufacturer’s protocol. The slides were scored for intratumoral Foxp3 expression density by consensus among two of three observers (AV, MG, PD). Blinded observers independently counted the number of regions with high density of Foxp3-expressing nuclei. Small discrepancies in cell counting were jointly reviewed and consensus was agreed upon. One photo (×200) was taken per slide of the area with the highest Foxp3 staining density. The cell count in each photo was multiplied by the number of densely populated regions to create a consistent approximate for the number of Foxp3-expressing cells in a single tumor slice. Cells at the tumor-invading stroma or in stromal folds were not counted; only tumor-infiltrating Tregs were included. Rat anti-mouse CD8 (Invitrogen #14-0808-82), rabbit anti-mouse F4/80 (Cell Signaling Technology #70076), rabbit anti-mouse Ly6G (Cell Signaling Technology #87048) and rabbit anti-mouse NK1.1 (Cell Signaling Technology #39197) staining was performed according to manufacturer’s protocol. For Ly6G, F4/80 and NK1.1, the intratumoral range of highest and lowest intensities (and intermediate categories) was proportionately scored. For Cd8 stains, we scored the tumor body: total number of positive cells in three representative high-power fields of 200×, and also scored the proportion of positive Cd8 cells in the invasive front (leading edge) of the tumor. For Foxp3+stains in the spleen, we selected three central red pulp areas and counted the positive staining cells (online supplemental figure 7).

### Immunofluorescence

Multiplexed tyramide signal amplification (TSA) immunofluorescence staining was performed using the BOND RX automated platform (Leica Microsystems). 4 µm sections of FFPE tumors were cut and mounted on charged slides. Dewaxing and heat-induced epitope retrieval of slides was automated on the Bond RX, using epitope retrieval solution 2 (Leica Microsystems, AR9640) for 20 min at 98°C. Using the Research Detection System 2 (Leica Microsystems, DS9777), endogenous peroxidase was blocked using 3% v/v hydrogen peroxide (VWR, 23622.260) in Tris Buffer Saline with Tween 20 (TBST) (VWR, J77500.K8) for 10 min and the slides further blocked with 10% w/v casein (Vector, SP5020) in TBST. Sections were then incubated with the primary antibody in Bond Antibody Diluent (Leica Microsystems, AR9352) for 30 min, followed by detection using Rat Impress (Vector MP7444) for 30 min, followed by a specific premixed TSA reagent (Perkin Elmer; 1:200 dilution) for 30 min. The first staining round used rat anti-mouse FOXP3 antibody (Invitrogen 14-5773-82; 1:100 dilution) and TSA570 (FP1488001KT). The second round used rat anti-mouse CD4 (Invitrogen 14-9766-82; 1:100 dilution) and TSA650 (FP1496001KT). Following labeling with TSA, antibodies were removed using a heat stripping step in epitope retrieval solution 1 for 15 min at 98°C. Finally, nuclei were counterstained with 0.33 µg/mL DAPI (Thermo Fisher, 62248) for 15 min and cover slipped with ProLong Gold Antifade Mountant (Thermo Fisher, P36930). Quantification of Treg immunofluorescence was performed using the HALO software (Indica Labs, V.3.6.4134.314). Analysis was performed using the Object Colocalization Fl V.2.1.4 algorithm to determine the number of cells positive for both Foxp3 (Cy3) and CD4 (Cy5). Results were calculated as the count of DAPI nuclei positive for both markers and normalized to the total area of the tumor analyzed.

### Statistical analysis

Statistical analysis of immune populations by flow cytometry was performed using a non-parametric two-tailed t-test (Mann-Whitney). For the subcutaneous tumor growth analysis of MC38 and Yumm2.1, a one-way analysis of variance (mixed-effects model; Tukey’s multiple comparison tests) was performed to compare the mean of each group with the mean of every other group. For survival analysis, a log-rank Mantel-Cox test was performed to compare the survival curves between groups. For the Treg analysis in the peripheral blood, using the GTEx dataset, a non-parametric one-tailed t-test (Mann-Whitney) was performed. For all analysis, a p value<0.05 was considered significant. All statistical analysis was performed using GraphPad Prism V.9.

### Study approval

All procedures involving animals were performed under the Home Office approved project licence PPL PP0466403, and UK Home Office regulations under the Animals (Scientific Procedures) Act 1986. The studies received ethical approval by the Cancer Research UK Manchester Institute’s Animal Welfare and Ethics Review Body.

## Supplementary material

10.1136/jitc-2025-012527 online supplemental material 1

10.1136/jitc-2025-012527 online supplemental material 2

10.1136/jitc-2025-012527 online supplemental material 3

10.1136/jitc-2025-012527 online supplemental material 4

10.1136/jitc-2025-012527 online supplemental material 5

10.1136/jitc-2025-012527 online supplemental material 6

10.1136/jitc-2025-012527 online supplemental material 7

10.1136/jitc-2025-012527 online supplemental material 8

10.1136/jitc-2025-012527 online supplemental material 9

10.1136/jitc-2025-012527 Uncited online supplemental material 10

## Data Availability

All data relevant to the study are included in the article or uploaded as supplementary information.
