# Supplementary material for: Systemic immunosuppression from ultraviolet radiation exposure inhibits cancer immunotherapy
Source: J Immunother Cancer. 2025 Oct 31;13(10):e012527. doi: 10.1136/jitc-2025-012527 (PMC12581074; doi:10.1136/jitc-2025-012527)
Supplement: online supplemental material 1 [file jitc-13-10-s001.docx]

**Supplementary Materials**

**Figures S1-S5**

**Supplementary Figure 1.** Cutaneous effects of acute UVR exposure

**Supplementary Figure 2.** Gating myeloid and lymphoid FACS strategies

**Supplementary Figure 3.** Peripheral blood Tregs after long term UVR, tumour growth and animal survival, and transcription signature clusters from spatial transcriptomics

**Supplementary Figure 4.** Spatial transcriptomics analysis of Non-UVR, UVR control and Anti-PD1 tumours

**Supplementary Figure 5.** Characterization of tumour immune microenvironment in non-UVR and UVR MC38 tumours.

**Supplementary Figure 6.** Graphical abstract

**Supplementary Figure 7.** Immunohistochemistry scores of tumours.

**Table S1-S8**

**Supplementary Table 1:** Marker genes for spatial transcriptomics clusters of MC38 Non-UVR and UVR tumours

**Supplementary Table 2:** Anti-PD1 (Non-UVR vs UVR) tumour differential expression analysis from spatial transcriptomics

**Supplementary Table 3:** IgG2a (Non-UVR vs UVR) tumour differential expression analysis from spatial transcriptomics

**Supplementary Table 4:** Cell types enriched in Cluster 6 from spatial transcriptomics MC38 tumours

**Supplementary Table 5:** GSEA analysis of differentially expressed genes in MC38 IgG2A tumours (UVR versus Non-UVR)

**Supplementary Table 6:** Immunohistochemistry scores

**Supplementary Table 7:** GSEA analysis of differentially expressed genes in MC38 Anti-PD1 tumours (UVR versus Non-UVR)

**Supplementary Table 8:** Patient characteristics: age and sex in the GTEx cohort by level of sun damage score

**Supplementary Figures**

**
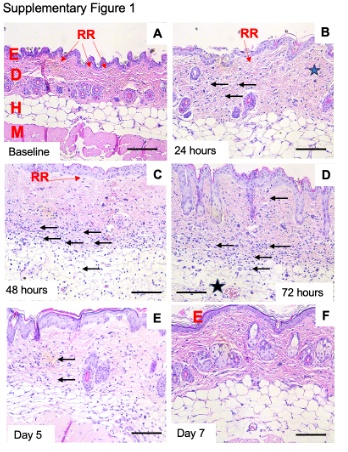
**

**Supplementary Figure 1. Cutaneous effects of acute UVR exposure**

**A**. Representative photomicrograph of haematoxylin and eosin (H&E) stain of non-irradiated mouse back skin showing epidermis (E, red) with bilayer of epidermal keratinocytes, dermis (D, red) and pronounced rete ridge folding (RR, red and red arrows), hypodermis (H, red) and subcutaneous muscular layer (M, red). **B**. Representative photomicrograph of H&E skin 24 hours after 6SED UVR exposure showing flattening of rete ridges (RR, red and red arrows), separation of the collagen dermal bundles (oedema, blue star), incipient inflammatory cell infiltrate within dermal collagen bundles (black arrows). **C.** Representative photomicrograph of H&E skin 48 hours after 6SED UVR exposure showing thickening of the epidermis, dermis and hypodermis, pronounced inflammatory cell infiltrate in the dermis and hypodermis (black arrows) and flattening of the rete ridges (RR red and red arrow). **D.** Representative photomicrograph of H&E skin 72 hours after 6SED UVR exposure showing thickening of the epidermis, dermis and hypodermis, pronounced inflammatory cell infiltrate in the dermis and hypodermis (black arrows) and vasodilation in the dermis with swollen endothelial cells (black star).  **E.**  Representative photomicrograph of H&E mouse skin 5 days after 6SED UVR exposure showing resolving inflammation in the dermis and hypodermis (black arrows). **F.** Representative photomicrograph of H&E UVR skin 7 days after UVR exposure showing increased layers in the epidermis (E, red), flattened RR and resolved inflammatory response in the dermis. Scale bars: 150 um.

**
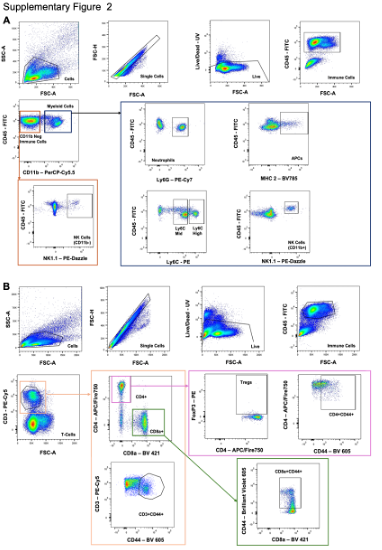
**

**Supplementary Figure 2. Gating myeloid and lymphoid FACS strategies**

A filtered sequential selection approach was used to identify each subpopulation. **A.** Myeloid panel. Live single cells were firstly selected based on CD45 expression. All CD45^+^ cells (Immune Cells) were then filtered according to CD11b expression. CD45^+^CD11b^-^ cells (CD11b Neg Immune Cells) were subsequently analysed based on expression of NK1.1 marker to identify the NK Cells (CD11b^-^) population. CD45^+^CD11b^+^ cells (Myeloid cells) were further subdivided into Neutrophils, APCs, Ly6C Mid, Ly6C High and NK Cells (CD11b^+^) according to the expression of Ly6G, MHCII, Ly6C and NK1.1, respectively. **B.** Lymphoid panel. Live single cells were firstly selected based on CD45 expression. All CD45^+^ cells (Immune Cells) were then filtered according to CD3 expression. All CD45^+^CD3^+^ cells (T Cells) were then subdivided into CD4^+^, CD8a^+^ and CD3^+^CD44^+^ according to the expression of CD4, CD8a and CD44 markers, respectively. CD4^+^Foxp3+ cells (Tregs) were gated on the CD4 population. CD4^+^ CD44^+^ cells were gated on the CD4 population. CD8a^+^CD44^+^ cells were gated on the C88a population.

**
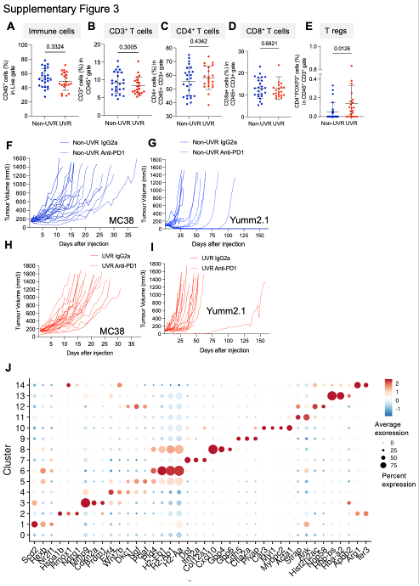
**

**Supplementary Figure 3. Peripheral blood Tregs after long term UVR, tumour growth and animal survival, and transcription signature clusters from spatial transcriptomics**

**A.** CD45^+^ cells (%) in Live gate, **B.** CD3^+^ cells (%) in CD45^+^ gate, **C.** CD4^+^ cells (%) in CD45^+^CD3^+^ gate, **D.** CD8a^+^ cells (%) in CD45^+^CD3^+^ gate, **E.** CD4^+^FOXP3^+^ cells (%) in CD45^+^CD3^+^ gated immune cell populations by flow cytometry in the peripheral blood of UVR mice exposed to 20 weeks of UVR (n=21) and Non-UVR (n=25) matched controls. Whisker graphs depict individual values, medians and interquartile ranges, Mann-Whitney two-tailed tests. **F.** Individual tumour growth curves of MC38 Non-UVR IgG2a and MC38 Non-UVR Anti-PD1, **G.** MC38 UVR and MC38 UVR Anti-PD1, **H.** Yumm2.1 Non-UVR IgG2a and Yumm2.1 Non-UVR Anti-PD1 and **I.** Yumm2.1 UVR and Yumm2.1 UVR Anti-PD1. MC38: Non-UVR IgG2a n=12; MC38: Non-UVR Anti-PD1 = 11; n MC38 UVR IgG2a = 12; n MC38: UVR Anti-PD1 = 11; Yumm2.1: Non-UVR IgG2a n=9; Yumm2.1: Non-UVR Anti-PD1 n=10; Yumm2.1: UVR IgG2a n=10; Yumm2.1: UVR Anti-PD1 n=10. **J.** Expression of top three marker genes for each transcriptional signature cluster in all MC38 Non-UVR and UVR spatially analysed tumours (related to Figure 3), red represents high expression, blue represents lower expression, size of dots represents percent of cluster expressing marker genes.

**
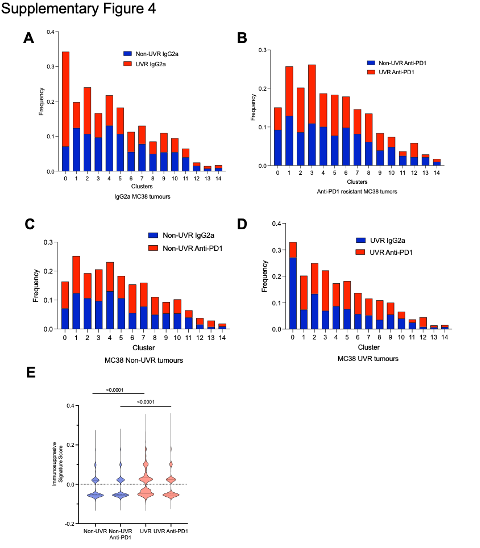
**

**Supplementary Figure 4. Spatial transcriptomics analysis of Non-UVR, UVR control and Anti-PD1 tumours**

**A.** Frequency of each cluster in MC38 Non-UVR IgG2a (blue) and UVR IgG2a (red) tumours; **B.** Frequency of each cluster in MC38 Non-UVR Anti-PD1 (blue) and UVR Anti-PD1 (red) tumours; **C.** Frequency of each cluster in MC38 Non-UVR IgG2a (blue) and Non-UVR Anti-PD1 (red) tumours; **D.** Frequency of each cluster in MC38 UVR IgG2a (blue) and UVR Anti-PD1 (red) tumours. **E.** Quantification of immunosuppressive signature from spatial transcriptomic analysis of MC38 Non-UVR (blue) and UVR (red) tumours grouped by treatment. Data represents signature score in individual spots across tumours, Mann Whitney U two sided.

**
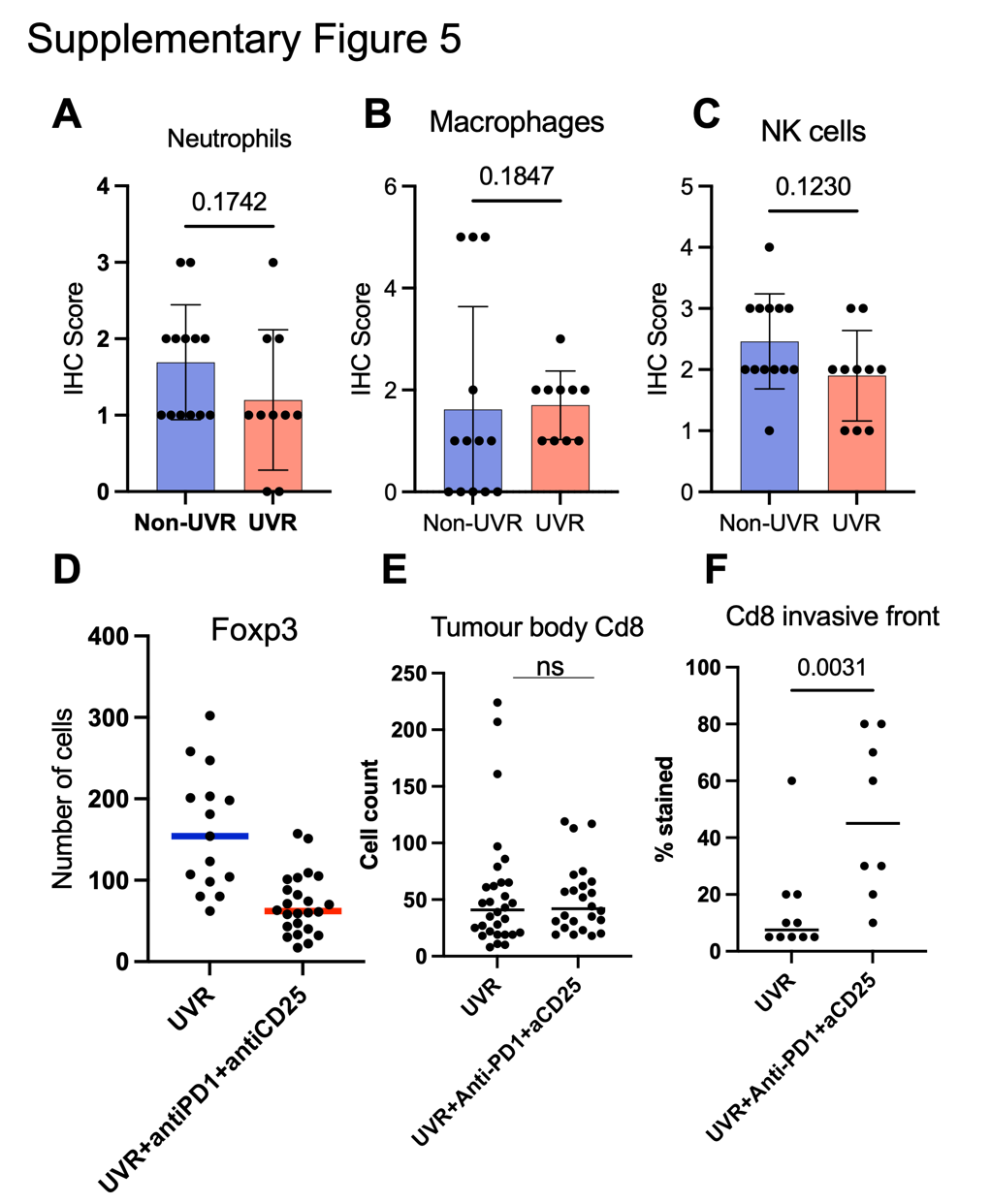
**

**Supplementary Figure 5. Characterization of tumour immune microenvironment in non-UVR and UVR MC38 tumours.**

Immunohistochemistry counts of **A.** LysG^+^ cells **B.** F4/80^+^ cells and **C.** NK1.1^+^ cells in MC38 Non-UVR (n=6) and UVR (n=5) tumours and Yumm2.1 Non-UVR (n=7) and UVR (n=5) mice, Mann-Whitney test. **D.** FoxP3+ cells in spleens of Yumm2.1 and MC38 UVR (n=13) tumours and Yumm2.1, MC38 UVR-AntiPD1-AntiCD25 (n=8) tumours. **E.** Cd8+ cells in tumour body and **F.** % of cells that are Cd8+ in Yumm2.1 and MC38 UVR (n=13) tumours and Yumm2.1, MC38 UVR-AntiPD1-AntiCD25 (n=8) tumours.

**Supplementary Figure 7**


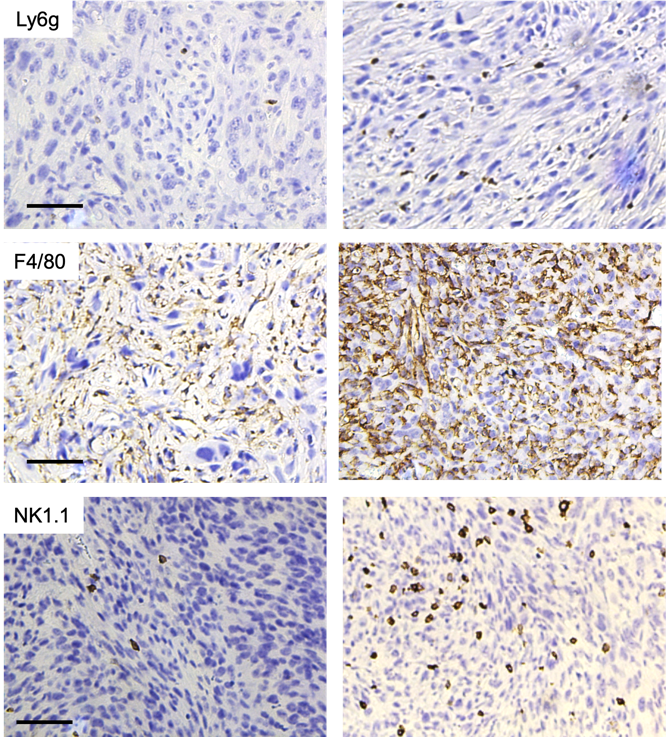


**Supplementary Figure 7. Immunohistochemistry scores of tumours.** Immunohistochemistry range staining for Ly6g, F4/80 and NK1.1, left column lowest range stain, Right column highest range stain. Bar: 30 um.

**Supplementary Tables**

**Supplementary Table 1**

Marker genes for spatial transcriptomics clusters of MC38 Non-UVR and UVR tumours

**Supplementary Table 2**

Anti-PD1 (Non-UVR vs UVR) tumour differential expression analysis from spatial transcriptomics

**Supplementary Table 3**

IgG2a (Non-UVR vs UVR) tumour differential expression analysis from spatial transcriptomics

**Supplementary Table 4**

Cell types enriched in Cluster 6 from spatial transcriptomics MC38 tumours

**Supplementary Table 5**

GSEA analysis of differentially expressed genes in MC38 IgG2A tumours (UVR versus Non-UVR)

**Supplementary Table 6**

Immunohistochemistry scores of LysG^+^ cells (neutrophils), F4/80^+^ cells (macrophages) and NK1.1^+^ cells (NK cells) in MC38 and Yumm2.1 UVR and non-UVR tumours.

**Supplementary Table 7**

GSEA analysis of differentially expressed genes in MC38 Anti-PD1 tumours (UVR versus Non-UVR)

**Supplementary Table 8**

Patient characteristics: age and sex in the GTEx cohort by level of sun damage score
