# Supplementary figures and images for: Systemic immunosuppression from ultraviolet radiation exposure inhibits cancer immunotherapy
Source: J Immunother Cancer. 2025 Oct 31;13(10):e012527. doi: 10.1136/jitc-2025-012527 (PMC12581074; doi:10.1136/jitc-2025-012527)

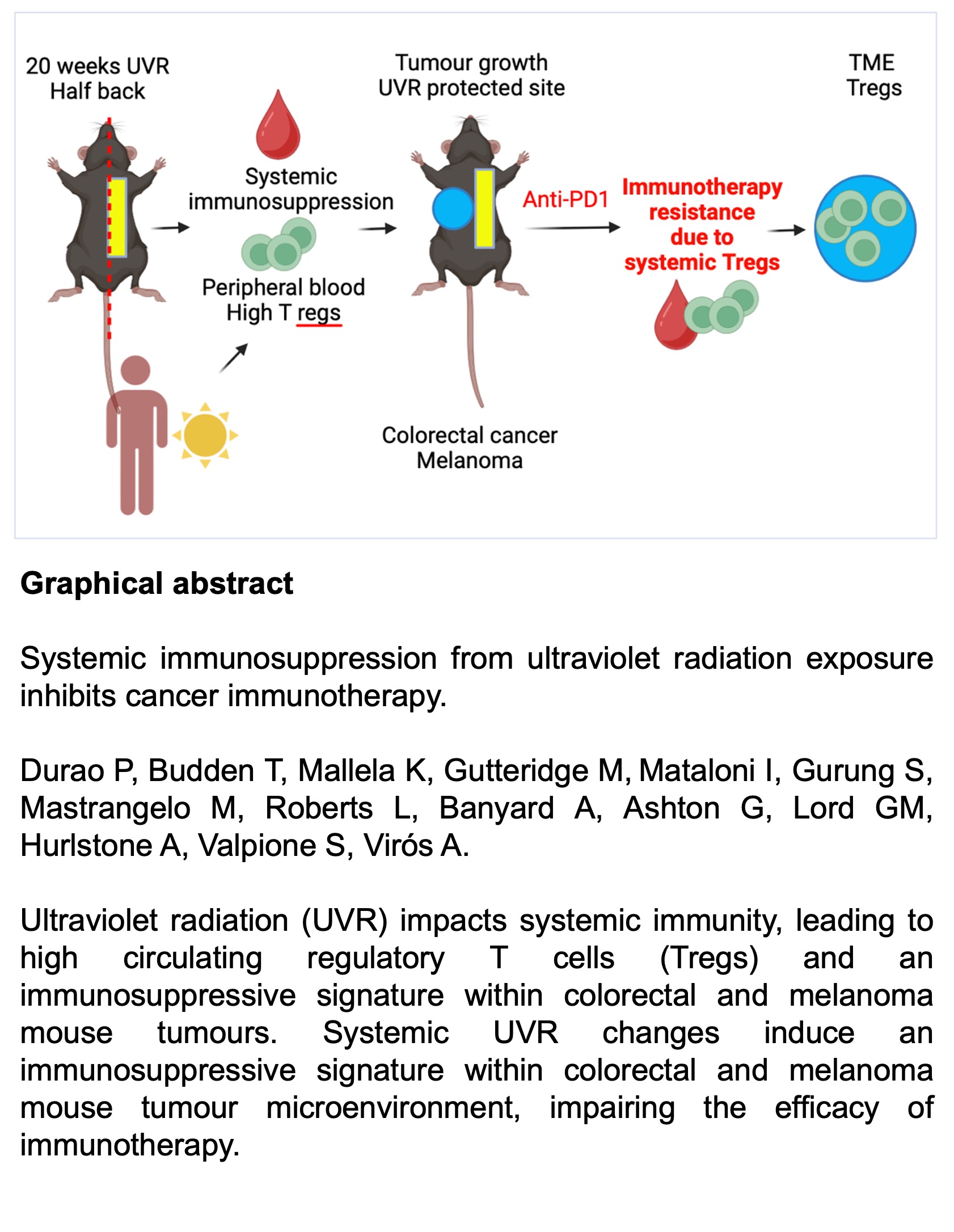

Supplement: Uncited online supplemental material 10 [file jitc-13-10-s010.jpg]
